# Seizures in 204 comatose children: incidence and outcome

**DOI:** 10.1007/s00134-012-2529-9

**Published:** 2012-04-11

**Authors:** Fenella J. Kirkham, Angela M. Wade, Fiona McElduff, Stewart G. Boyd, Robert C. Tasker, Melinda Edwards, Brian G. R. Neville, Norbert Peshu, Charles R. J. C. Newton

**Affiliations:** 1 Neurosciences Unit, UCL Institute of Child Health, London, UK; 2 Child Health, University hospitals Southampton, Southampton, UK; 3 Centre for Paediatric Epidemiology and Biostatistics, UCL Institute of Child Health, London, UK; 4 Neurophysiology Department, Great Ormond Street Hospital NHS Trust, London, UK; 5 Paediatric Intensive Care Unit, University of Cambridge, Addenbrooke’s Hospital, Cambridge, UK; 6 Harvard Medical School, Children’s Hospital, Boston, USA; 7 Evelina Children’s Hospital, Guy’s and St Thomas’ NHS Trust, London, UK; 8 Centre for Geographic Medical Research (Coast), Kenya Medical Research Institute, Kilifi, Kenya

**Keywords:** Seizures, Status epilepticus, Coma, Child, Outcome

## Abstract

**Purpose:**

Seizures are common in comatose children, but may be clinically subtle or only manifest on continuous electroencephalographic monitoring (cEEG); any association with outcome remains uncertain.

**Methods:**

cEEG (one to three channels) was performed for a median 42 h (range 2–630 h) in 204 unventilated and ventilated children aged ≤15 years (18 neonates, 61 infants) in coma with different aetiologies. Outcome at 1 month was independently determined and dichotomized for survivors into favourable (normal or moderate neurological handicap) and unfavourable (severe handicap or vegetative state).

**Results:**

Of the 204 patients, 110 had clinical seizures (CS) before cEEG commenced. During cEEG, 74 patients (36 %, 95 % confidence interval, 95 % CI, 32–41 %) had electroencephalographic seizures (ES), the majority without clinical accompaniment (non-convulsive seizures, NCS). CS occurred before NCS in 69 of the 204 patients; 5 ventilated with NCS had no CS observed. Death (93/204; 46 %) was independently predicted by admission Paediatric Index of Mortality (PIM; adjusted odds ratio, aOR, 1.027, 95 % CI 1.012–1.042; *p* < 0.0005), Adelaide coma score (aOR 0.813, 95 % CI 0.700–0.943; *p* = 0.006), and EEG grade on admission (excess slow with >3 % fast, aOR 5.43, 95 % CI 1.90–15.6; excess slow with <3 % fast, aOR 8.71, 95 % CI 2.58–29.4; low amplitude, 10th centile <9 µV, aOR 3.78, 95 % CI 1.23–11.7; and burst suppression, aOR 10.68, 95 % CI 2.31–49.4) compared with normal cEEG, as well as absence of CS at any time (aOR 2.38, 95 % CI 1.18–4.81). Unfavourable outcome (29/111 survivors; 26 %) was independently predicted by the presence of ES (aOR 15.4, 95 % CI 4.7–49.7) and PIM (aOR 1.036, 95 % CI 1.013–1.059).

**Conclusion:**

Seizures are common in comatose children, and are associated with an unfavourable outcome in survivors. cEEG allows the detection of subtle CS and NCS and is a prognostic tool.

## Introduction

Seizures may be difficult to detect in unconscious children, particularly if the episode is partial or subtle or when neuromuscular blocking (NMB) agents are used during mechanical ventilation [1]. Electroencephalography (EEG) may be helpful in this situation, but there are a range of possibilities when observing electroencephalographic seizure (ES) activity. For example, in the child who is able to move, if there is no convulsive activity, then the state could be defined as non-motor, non-convulsive seizures (NCS). Alternatively, there may be subtle clinical features of epileptic nystagmus or facial myoclonus [2–4]. Monitoring with continuous EEG (cEEG) in neonates and older children is sensitive for detection of ES, with few false-positives [5–7]. ES may be frequent, with a prolonged time-course [8–16], but their significance is unknown, so it not clear whether cEEG is advisable or anticonvulsant treatment should be given for subtle seizures, NCS and/or ES in comatose patients or those receiving NMB agents. In order to answer this question, we need to know whether there is a relationship between these events and eventual outcome, independent of underlying aetiology.

The purpose of this study was to examine the prevalence of ES in comatose children and to determine any association of subtle seizures or ES with mortality and morbidity.

## Methods

### Ethical approval

The research ethics committees of Guys Hospital Medical School and the Kenyan Medical Research Institute gave ethical approval for the work undertaken in their respective institutions. At the time of this observational study, individual written informed consent was not required to report clinically indicated monitoring or follow-up, but the investigators discussed the research with parents or guardians and obtained verbal consent to monitor EEG activity and to follow-up the patients as part of clinical care.

### Study populations

Two prospectively recruited consecutive cohorts of unconscious children without previous acute neurological injury underwent cEEG monitoring. In the first (Guy’s Hospital, London) all of the children were mechanically ventilated and received pancuronium for NMB. In the second (Kenya Medical Research Institute, Kilifi, Kenya) none of the children underwent mechanical ventilation since facilities were not available, and so they could be assessed clinically. Any child older than 1 month with an acute encephalopathy and a summated Adelaide coma score (ACS) [17] less than normal for age was eligible, and as there was sufficient monitoring equipment available, all had cEEG monitoring until they were able to localize a painful stimulus (>9 months) or flex to pain (<9 months) or death.

#### Clinical observations

Diagnosis followed standard definitions (see “Aetiology of coma”). In unventilated patients, level of consciousness was assessed on admission and then 6-hourly using the ACS until the child was drinking. In ventilated patients, ACS was obtained on admission. Thereafter, clinical examination or detection of clinical seizures (CS) was usually precluded by the use of medication for sedation and NMB. In all patients, clinical signs suggestive of seizures, such as jerking, or increases in blood pressure or heart rate, were noted. In patients without mechanical ventilation, if ES were noted on the cEEG, the bedside attendant undertook a careful clinical examination. Evidence of subtle CS (e.g. changes in pupil response, blood pressure, or heart rate, or clonic movements of the digits, face or eyes) was recorded.

#### Electroencephalography

**Fig. 1:**
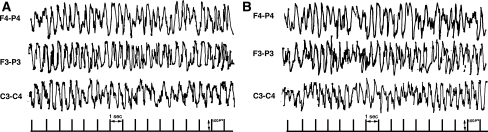
Oxford Medilog trace from a girl aged 4 years with cavernous sinus thrombosis and a cardiac arrest showing a very prolonged ES, which commenced at 0130 hours (**a**). The discharges continued unrecognized by the nursing staff until 0500 hours (**b**). Subsequently the EEG became isoelectric and the child died a brain death

**Fig. 2:**
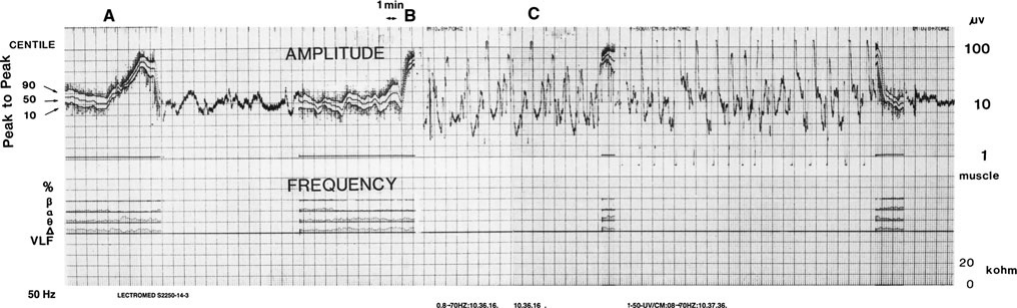
CFAM trace from a baby aged 3 months with Reye’s syndrome showing a sudden increase in amplitude of compressed trace at *A* and *B* associated with a rhythmical seizure discharge on the raw EEG playout at *C*. The main graticules, when the trace is being written, are at intervals of 0.5 cm

Two EEG devices and montages were used in these studies. In 52 patients managed at Guy’s hospital, the output from a three-channel Oxford Medilog EEG machine [14, 15] with disc electrodes placed at F4–P4 and F3–P3 and C3–C4 on the International 10–20 system (reference electrodes just behind the hairline) was displayed in real time at the bedside (Siemens monitor, Erlangen, Germany) and the raw EEG was replayed off-line through a commercial system (Oxford Systems, Oxford, UK). This equipment did not include amplitude and frequency integration, and staff found it difficult to recognize seizures (Fig. 1) in real time. Therefore, in the remaining 152 patients, including all 46 Kenyans, a one- or two-channel cerebral function analysing monitor (CFAM) [7, 8] was used, calibrated using a conventional multichannel EEG. The disc electrodes were placed at P3–P4 (reference electrode just behind the hairline) for single channel monitoring (*n* = 118), or P3–P4 and F4–P4 for bilateral monitoring (*n* = 34) with data displayed on a paper trace with raw EEG displayed automatically at least every 10 min. A button could be pressed by attending staff to record raw EEG during increases in amplitude representing possible seizures (Fig. 2). Electrode impedance was maintained below 2 kOhm and mains artefact as low as possible [11, 14].

### Clinical management of seizures

The attending physician decided on the prescription of prophylactic or treatment doses of antiepileptic drugs. Rectal diazepam (0.5 mg/kg) or paraldehyde (0.2 ml/kg) was given to stop seizures (CS or ES) lasting longer than 5 min. Some children were prescribed prophylactic parenteral phenytoin (18 mg/kg) or phenobarbital (15–20 mg/kg) before cEEG commenced. Others received these anticonvulsants only when there was more than one CS or ES. If the episode continued, then repeat doses were given after 6–12 h.

### Severity of illness and outcome assessment

The Paediatric Index of Mortality (PIM) [18] and ACS on admission were recorded. There was active surveillance for death during the study. Treatment was only withdrawn in the event of brain death. All children who survived initial hospitalization were followed-up at 1 month after admission to hospital by one of the paediatric neurologists in the study, who undertook a detailed history from the parents about any change from premorbid cognition and behaviour, as well as a detailed neurological examination. Using this information, outcome was classified on the Glasgow Outcome Scale by an investigator (B.G.R.N.) blinded to the admission clinical and cEEG data. Outcome was dichotomized: children who remained in a vegetative state, or had a movement disorder precluding ambulation, or severe learning difficulties, were classified as having an unfavourable outcome, with the remainder as having a favourable outcome. Where possible, and always if the neurological examination was abnormal, defined as reduced level of consciousness, or if abnormalities of motor, visual, auditory or cognitive function were found, further follow-up was arranged and longer term outcome assessed using the same methodology.

### Data and statistical analyses

#### Analysis of ES episodes

An ES was defined as the presence of rhythmical spike-and-wave activity for more than 15 s, and was considered subclinical if there were no clinical manifestations. For the CFAM data, all traces were analysed independently for the presence of ES by S.G.B. and F.J.K.; consensus was reached if there was any disagreement. The number and total duration of ES activity, and duration of the longest ES, were obtained from the traces.

#### Statistical analyses

Differences in demographics and outcome measures between the three groups (UK neonates, UK infants and children, Kenyan infants and children) were tested using one-way parametric or Kruskal-Wallis ANOVA or the chi-squared test as appropriate. The hypothesis test for proportions was used to compare prevalences. Logistic regression models were used to investigate potential predictors of two outcomes: death in the first month and unfavourable outcome at 1 month amongst the survivors, including clinically important potential predictors: cohort (UK or Kenya), aetiology, PIM score, ACS and EEG grade on admission, prophylactic anticonvulsant administration with benzodiazepines and/or phenobarbitone, CS at any time, and the presence (yes/no), number, total duration and single longest duration of ES. For prediction of unfavourable outcome, the duration of EEG monitoring was also explored as a potential confounder. Unadjusted and adjusted odds ratios (OR) with 95 % confidence intervals (CI) are presented in the tables. Cerebral malaria was chosen as the reference because seizures and coma are common, while outcome is variable, although the malaria parasite does not directly invade the brain parenchyma. Variables were added to multivariable models according to statistical significance in a forward step-wise fashion. All variables retained led to a significant change in log likelihood. Variables not initially significant were investigated after adjustment for other factors. The significance level for inclusion was primarily set at *p* < 0.05.

## Results

### Aetiology of coma

Aetiological categories included: cerebral malaria (defined as *Plasmodium falciparum* asexual parasitaemia in a normoglycaemic patient unable to localize a painful stimulus at least 6 h after last seizure [19]), meningitis (bacteria identified in the cerebrospinal fluid by microscopy, culture or antigen), encephalitis (febrile illness with previous viral infection or pleocytosis and no bacteria), Reye-like syndrome (coma with high blood ammonia), hypoxic–ischaemic encephalopathy (witnessed cardiopulmonary arrest), head injury (evidence of trauma to the head), hypertensive encephalopathy (coma with severe hypertension), thrombotic thrombocytopoenic purpura/haemolytic uraemic syndrome (coma associated with fragmented or burr red cells and thrombocytopaenia), systemic vasculitis (coma associated with laboratory findings diagnostic of autoimmunity), intracerebral haemorrhage (spontaneous coma with intracerebral bleeding on CT), drug toxicity and cavernous sinus thrombosis (Table 1) .

**Table 1:** Characteristics of UK and Kenyan populations comprising in total 204 comatose neonates, infants and children

	UK neonates (*n* = 18)	UK infants and children (*n* = 140)	Kenyan infants and children (*n* = 46)
Boys	11 (61 %)	71 (51 %)	24 (52 %)
Age (years)			
Median	0.01	2.0	2.7
Range	0–0.08	0.1–15	0.25–7
Aetiology			
Cerebral malaria	0	0	42
Meningitis	0	6	3
Encephalitis	0	10	1
Reye-like syndrome	0	3	0
Hypoxic–ischaemic	18	96	0
Head injury	0	18	0
Hypertensive encephalopathy	0	1	0
Intracerebral haemorrhage	0	1	0
Cavernous sinus thrombosis	0	1	0
Drug toxicity (toluene)	0	1	0
Systemic vasculitis	0	1	0
Thrombotic thrombocytopenic purpura/haemolytic uraemic syndrome	0	2	0
Paediatric index of mortality			
Median	13.1	56	12.8
Range	2.8–91.3	23.6–97.9	7.5–30.4
Initial summated ACS			
Median	3	4	7
Range	3–12	3–12	3–12
Clinical seizures witnessed prior to monitoring	11 (61 %)	57 (41 %)	42 (91 %)
Type of EEG recording			
Oxford Medilog	7	45	0
CFAM	11	95	46
Duration of EEG monitoring (h)			
Median	60	48	12
Range	6–120	4–630	2–90
EEG grade on admission			
Normal	3	30	6
Excess slow with >3 % fast	7	37	26
Excess slow with <3 % fast	1	20	11
Low amplitude (10th <9 µV)	4	30	3
Burst suppression	2	15	0
Isoelectric	1	8	0
Electroencephalographic seizures (ES) (%)	8 (44 %)	49 (35 %)	17 (37 %)
Number of ES seizures			
Median	7	28	7
Range	3–137	1–531	1–46
Total duration of ES (min)			
Median	44	135	63
Range	10–517	2–2,358	6–290
Duration of longest ES (min)			
Median	9	18	16
Range	4–24	2–1,440	1–232
Survived to 1 month			
No. of patients	11	60	40
Percent of total	61 (95 % CI 39, 80)	43 (95 % CI 35, 51)	87 (74, 94)
Unfavourable neurological outcome at 1 month			
No. of patients	1	23	5
Percent of survivors at 1 month	9 (95 % CI 16, 38)	38 (95 % CI 27, 51)	13 (95 % CI 6, 26)

### The prevalence of ES activity during coma

Overall, ES were detected in 74 (36 %; 95 % CI 30, 43 %) of the 204 comatose children with cEEG. The number of seizures and total duration of seizures correlated with total duration of monitoring (*R* ^2 ^= 0.09, *p* = 0.0005; *R* ^2 ^= 0.05; *p*=0.001, respectively). ES occurred in 69 (62.7 %) of 110 patients with CS compared with 5 (5.3 %) of 94 with no CS witnessed (difference 5.7 %, 95 % CI 4.6, 66.4 %; *p* = 0.0005). There was no difference between the 158 ventilated patients receiving NMB agents (36 %, 95 % CI 29, 44 %) and the 46 non-ventilated children (37 %, 95 % CI 26, 51 %; *p* = 0.5), between the neonates (44 %, 95 % CI 25, 66 %) and the older children (36 %, 95 % CI 29, 43 %; *p* = 0.5) or between the seizures detected with the CFAM (38 %, 95 % CI 31, 46 %) and with the Oxford Medilog (31 %, 95 % CI 20, 44%; *p* = 0.2).

In 46 Kenyan children who were not mechanically ventilated, cEEG was undertaken for a median of 12 (range 2–90) h (Table 1). CS were seen in 42 (91 %) and at least one ES was seen in 17 (40 %, 95 % CI 26, 57 %). ES were only present in those patients who had CS. The types of CS noted were generalized tonic–clonic seizures (*n* = 7), and only subtle seizures (e.g. clonic jerking of the fingers or epileptic nystagmus) in the other 35 patients. The median number of ES per patient was 7 (range 1–46). In 15 of the 46 children (33 %, 95 % CI 20, 48 %), these episodes occurred without any clinical concomitants (i.e. NCS). Of the 17 children with ES, 15 (88 %, 95 % CI 64, 99 %) also exhibited CS at some time. NCS were not observed as the sole form of seizure activity in any individual.

In 140 unconscious mechanically ventilated infants and children with a variety of diagnoses (Table 1), cEEG monitoring was carried out for a median of 48 h (range 4–630 h) commencing, on average, within 3 h of admission to the PICU. Before admission and the use of NMB agents, 57 of the 140 patients (41 %, 95 % CI 33, 49 %) had CS. In 11, ES activity was associated with subtle clinical events, e.g. clonic finger jerking. In contrast to the spontaneously breathing subgroup, ES were found in 5 of 83 patients (6 %, 95 % CI 2, 14 %) in whom CS had not been observed before or during admission.

Before prescription of NMB agents, 11 of 18 neonates with cardiac disease (61 %, 95 % CI 39, 80 %) had CS, 8 of whom (73 %, 95 % CI 43, 90 %) also had ES (Table 1), while ES were not detected in any others.

### Outcome and significance of ES activity during coma

Of the 204 patients, 93 (46 %, 95 % CI 39, 52 %) had died by 1 month. Mortality was higher in the infants and children requiring mechanical ventilation for life support (Table 2). In univariable logistic regression, in addition to absence of CS at any time, death was associated with the following findings on admission: aetiology, higher PIM score, lower ACS and lower cEEG grade (Table 2). These five variables were entered into the multivariable model. PIM score, ACS and EEG grade on admission as well as absence of CS at any time were associated with death (Table 2) in multivariable logistic regression after adjustment for aetiology. Anticonvulsant prophylaxis was not associated with mortality.

**Table 2:** Logistic regression for prediction of death in the first month

Variable	Survivors (*n* = 111)	Deaths (*n* = 93)	Odds ratios			
			Unadjusted	95 % CI	Adjusted	95 % CI
Age (years)						
Median	2	1.5	0.99	0.92–1.07		
Range	0–14	0–15				
Age group						
Neonate	11	7	0.74	0.28–2.0		
Older child	100	86				
Sex						
Male	61	45	1.3	0.75–2.26		
Female	50	48				
Cohort						
UK	71	87	8.17	3.28–20.4		
Kenya	40	6				
Aetiology						
Cerebral malaria	38	4	1.0	–		
Hypoxic–ischaemic	45	69	14.6	4.9–43.6		
Head injury	10	8	7.6	1.9–30.4		
Encephalitis	10	1	0.97	0.10–9.50		
Meningitis	4	5	11.9	2.24–63.1		
Reye’s syndrome	2	1	4.75	0.35–64.7		
Other	2	5	23.8	3.43–164.7		
On admission						
PIM score						
Median	30.4	56.5	1.032	1.020, 1.044		
Range	3.7, 97.9	2.8, 95.7				
ACS						
Median	6	3	0.71	0.62–0.82		
Range	3–12	3–12				
cEEG						
Normal	31	8	1.0	–		
Excess slow with >3 % fast	43	27	2.43^a^	0.98–6.1^a^		
Excess slow with <3 % fast	14	18	5.0^a^	1.75–14.2^a^		
Low amplitude (10th <9 µV)	19	18	3.67^a^	1.33–10.1^a^		
Burst suppression	4	13	12.6^a^	3.22–49.3^a^		
Isoelectric	0	9	–	–		
During admission						
Presence of ES						
Yes	45	29	0.67	0.37–1.19		
No	66	64				
Number of ES						
Median	0	0	0.993	0.984–1.001		
Range	0–531	0–109				
Duration of ES (min)						
Median	0	0	1.000	0.999–1.001		
Range	0–2,358	0–1,655				
Duration of longest seizure (min)						
Median	0	0	1.001	0.999–1.003		
Range	0–360	0–1,440				
At any time						
Clinical seizures						
Yes	74	36	0.32	0.18–0.56		
No	37	57				
Benzodiazepine before monitoring						
Yes	18	13	0.84	0.39–1.72		
No	93	80				
Phenytoin or phenobarbital before monitoring						
Yes	11	9	1.03	0.41–2.60		
No	100	84				
PIM score					1.027	1.012, 1.042
ACS					0.813	0.700, 0.943
cEEG						
Normal					1.0	–
Excess slow with >3 % fast					5.43^a^	1.90, 15.6^a^
Excess slow with <3 % fast					8.71^a^	2.58, 29.4^a^
Low amplitude (10th <9 µV)					3.78^a^	1.23, 11.7^a^
Burst suppression					10.68^b^	2.31, 49.4^a^
Isoelectric					–	–
Absence of CS at any time					2.38	1.18, 4.81

^a^Compared to normal EEG on admission

**Fig. 3:**
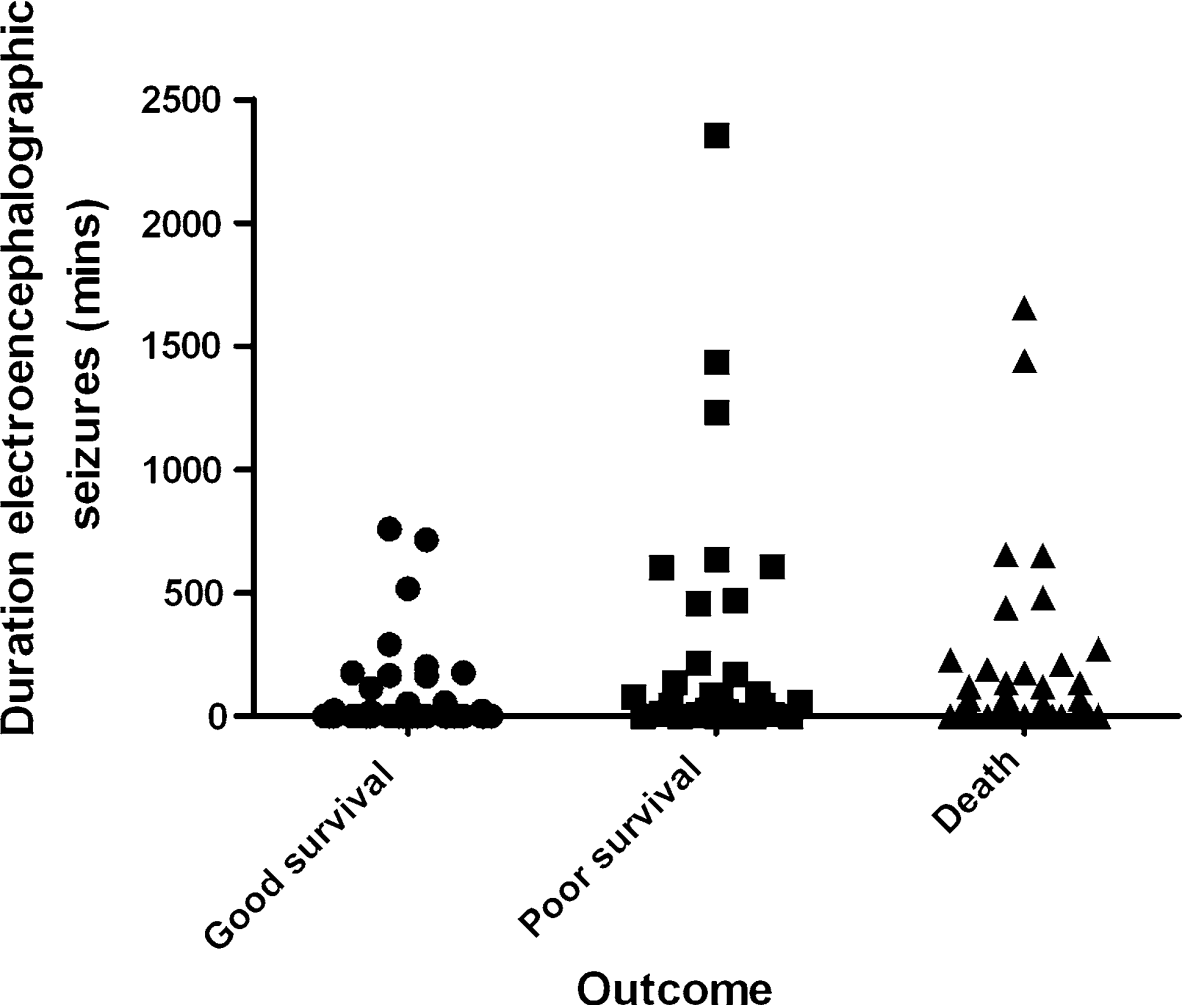
Duration of electrographic seizures in children who survived with good (normal or moderate disability) and poor (severe or vegetative state) outcome and in those who died

At 1 month, 29 patients (26 %, 95 % CI 19, 35 %, of the survivors; 14 %, 95 % CI 10, 20 %, of the total) were either severely handicapped or in a vegetative state. Outcome in survivors was worse (Table 3) in the ventilated cohort, in those with longer duration of monitoring, and in those with hypoxic–ischaemic aetiology, lower PIM score, lower cEEG grade, CS at any time, ES at any time, and larger number and longer duration of ES activity (Fig. 3) and longest duration ES. No child had a favourable outcome when there had been more than 139 seizures, or a total duration of ES of more than 759 min, or an individual ES of more than 360 min. There was no significant association with age, sex or the use of anticonvulsant prophylaxis. In the final model, the presence of ES and PIM score remained in the model for predicting unfavourable neurological outcome (Table 3) independent of cohort and duration of EEG monitoring. None of the other variables was statistically significant after taking these variables into account. Longer term outcome was available for all 71 UK survivors at a median of 6 months (range 1.5–144 months) and for 13 of 40 Kenyan survivors, including all five with an unfavourable outcome at 1 month, at a median of 12 months (range 1.5–40 months). Of the 29 with an unfavourable outcome at 1 month, on final follow-up at a median of 6 months (range 1.5–144 months), one had improved to moderate handicap, eight had died and the remainder stayed in the same outcome category. Although four of those with a favourable outcome at 1 month subsequently died, none deteriorated neurologically when assessed at a median age of 6 months (range 1.5–48 months).

**Table 3:** Odds ratios from logistic regression for prediction of unfavourable outcome in 111 survivors at 1 month

Variable	Good outcome (*n* = 82)	Unfavourable outcome (*n* = 29)	Odds ratios for unfavourable outcome			
			Unadjusted	95 % CI	Adjusted	95 % CI
Age	–	–	0.93	0.81–1.07		
Age group						
Neonate	10	1	0.26	0.03–2.1		
Older child	72	28				
Gender						
Male	47	14	1.44	0.62–3.37		
Female	35	15				
Cohort						
UK	47	24	3.57	1.24–10.3		
Kenya	35	5				
Duration of monitoring (h)						
Median	47	120	1.010	1.004, 1.016		
Range	2–276	9–264				
Aetiology						
Cerebral malaria	35	3	1.0	–		
Hypoxic–ischaemic	28	17	7.08	1.89–26.6		
Head injury	9	1	1.30	0.12–14		
Encephalitis	8	2	2.92	0.42–20.4		
Meningitis	0	4	–	–		
Reye-like syndrome	0	2	–	–		
Other	2	0	–	–		
On admission						
PIM score						
Median	26	52.1	1.034	1.015–1.053		
Range	3.7–97.3	7.5–97.9				
ACS						
Median	6	5	1.17	0.97–1.41		
Range	3–12	3–12				
cEEG						
Normal	26	5	1.0	–		
Excess slow with >3 % fast	34	9	3.33^a^	0.29–38.1^a^		
Excess slow with <3 % fast	11	3	11.0^a^	0.82–147.9^a^		
Low amplitude (10th <9 µV)	10	9	11.3^a^	1.05–122^a^		
Burst suppression	1	3	15.6^a^	1.33–182^a^		
Isoelectric	0	0	–	–		
During admission						
Presence of ES						
Yes	21	24	13.9	4.72–41.2		
No	61	5				
Number of ES						
Median	0	13	1.027	1.007–1.035		
Range	0–137	0–531				
Duration of ES (min)						
Median	0	78	1.004	1.001–1.006		
Range	0–759	0–2,358				
Duration of longest ES (min)						
Median	0	11	1.007	1.001–1.014		
Range	0–360	0–360				
At any time						
Clinical seizures						
Yes	49	25	4.21	1.34–13.2		
No	33	4				
Benzodiazepine before monitoring						
Yes	16	2	0.31	0.07–1.42		
No	66	27				
Phenytoin or phenobarbital before monitoring						
Yes	8	3	1.07	0.26–4.33		
No	74	26				
Presence of EEG seizures					15.4	4.7, 49.7
PIM score					1.036	1.013, 1.059

^a^Compared to normal EEG on admission

In a multivariable model for prediction of death which included ES, the odds ratio and confidence intervals changed only marginally. Adding the predictors of death to a multivariable model for prediction of outcome in survivors did not change the model and minimally altered the odds ratio and confidence intervals for the effect of ES.

## Discussion

In this study ES occurred in over one-third of comatose children, and the prevalence of ES was related to the presence of CS and the underlying aetiology. Our data on prevalence of ES is consistent with those of previous studies of CS in unconscious adults and children [20–32] and of ES in paediatric [10, 33, 34] and adult intensive care [35], but not that of a recent study which detected ES in only 7 % of patients [36]. Independently of aetiology, PIM, ACS and the presence of CS, normal background cEEG activity predicted survival, while the presence of ES activity predicted an unfavourable outcome in survivors, although, as in an adult study [37], good outcomes may be seen after very prolonged seizures.

Most of the children had CS before EEG monitoring, but five (2 %), all ventilated, had only ES, i.e. NCS. This is a similar proportion to those found in children and adults who were in nonconvulsive status epilepticus on EEG during the first 3 days after presentation in coma [9, 38]. Among the nonventilated children, all who had ES had CS. It would seem prudent to monitor EEG to detect NCS in children who remain unconscious or in children with an acute encephalopathy and CS requiring NMB agents for ventilation [9], or in the absence of a reliable history, e.g. after non-accidental injury.

These results must be interpreted cautiously. The criteria for admission to the high-dependency unit and PICU differed, although our cohort had a variety of diagnoses, so we could assess whether the effect of seizures was independent of aetiology. Since we monitored only one to three channels in these cohorts, a proportion of seizures may have been missed [5]. Patients with traumatic brain injury or intracranial haemorrhage secondary to arteriovenous malformation may have unilateral discharges, while those with status epilepticus in the context of polymerase-γ mutations [39] or posterior reversible encephalopathy syndrome [40] typically have occipital involvement. These conditions should be borne in mind when the choice of electrode placement for longer term single or dual channel EEG monitoring is decided. The use of cEEG should be evaluated by a comparison between standard EEG and cEEG at the first opportunity. Although detection of any seizure in a patient may not be better, two or more channels may detect more seizures, particularly in unilateral disease [41]. Further studies comparing one- and two-channel amplitude integrated displays with raw EEG are warranted but as seizure detection is the priority, there may be an advantage in setting up a simple single-channel amplitude integrated EEG monitor with raw EEG display on admission, as ES are detected in the first hour in the majority of patients with seizures [33]. In our data, there was no evidence that monitoring three channels with the Oxford Medilog had any advantage over monitoring one or two channels with the CFAM which displays raw EEG regularly and on demand in addition to the advantage of easier review in real time allowed by the amplitude integrated display [10]. The effect on outcome of ES not manifest as rhythmical spike-and-wave detectable with limited channel EEG monitoring should be the subject of prospective studies with multichannel cEEG. We found an association between CS with survival, at face value surprising but previously documented after head injury [42], perhaps because CS precipitate admission. ES did not predict death either, perhaps because death is related to acute intracranial hypertension independent of seizures. We were only able to determine outcome in all the children at 1 month, and the number of surviving patients was relatively small for multivariable logistic regression. Despite these limitations, our data demonstrate an association between ES and unfavourable neurological outcome in survivors independent of age, aetiology and cohort. Further prospective studies with long-term follow-up are required, however, before definitive recommendations on monitoring and treatment can be made.

Routine [3] or cEEG [9] with video recording detects NCS or nonconvulsive status epilepticus in at least one-third of PICU patients presenting with CSE. There is concern that ongoing ES activity is under-recognised in these patients, who are usually sedated and sometimes given NMB agents for ventilation [3, 9]. In children in coma, Jette et al*.* [9] suggested excluding NCS even if there are no CS, and our data provide further evidence for this recommendation. However, multichannel cEEG is labour-intensive and expensive, treatment protocols have not been defined, and there are few data on any association with neurological outcome in coma with acute symptomatic CSE. Further research looking at the reliability of one- or two-channel cEEG monitoring devices in diagnosing NCS is required as the technical issues are resolved and our data add to the evidence that outcome may be predicted on the PICU [10].
